# A Contemporary Report of Low-Dose-Rate Brachytherapy for Prostate Cancer Using MRI for Risk Stratification: Disease Outcomes and Patient-Reported Quality of Life †

**DOI:** 10.3390/cancers15041336

**Published:** 2023-02-20

**Authors:** Mira Patel, William Tyler Turchan, Christopher G. Morris, Dana Augustine, Tianming Wu, Aytek Oto, Gregory P. Zagaja, Stanley L. Liauw

**Affiliations:** 1 Department of Radiation Oncology, University of Chicago Medicine, Chicago, IL 60637, USA; 2 Department of Radiation Oncology, University of Florida, Gainesville, FL 32610, USA; 3 Department of Radiology, University of Chicago Medicine, Chicago, IL 60637, USA; 4 Department of Urology, University of Chicago Medicine, Chicago, IL 60637, USA

**Keywords:** prostate cancer, brachytherapy, MRI, quality of life

## Abstract

**Simple Summary:**

This study describes the outcomes of 149 men with prostate cancer treated at a single center with Iodine-125 low dose rate brachytherapy (also known as radioactive seed implant). A total of 98% of men were considered biochemically controlled 7 years after implant. Men without clear extra-prostatic extension of disease on MRI had very high rates of control, suggesting that MRI can be used to safely select men with “unfavorable intermediate risk” disease to be treated with an implant alone, rather than a combined course of external beam radiation with brachytherapy, as some guidelines recommend. Severe late side effects to the bladder and rectum were uncommon, and quality of life was well preserved, with mild changes in urinary and sexual health, particularly within the first 2 years after the implant. A table describing symptom distress over time is provided to help guide patient expectations regarding quality of life after brachytherapy.

**Abstract:**

Purpose: We examined a prospective consecutive cohort of low dose rate (LDR) brachytherapy for prostate cancer to evaluate the efficacy of monotherapy for unfavorable-intermediate risk (UIR) disease, and explore factors associated with toxicity and quality of life (QOL). Methods: 149 men with prostate cancer, including 114 staged with MRI, received Iodine-125 brachytherapy alone (144–145 Gy) or following external beam radiation therapy (110 Gy; EBRT). Patient-reported QOL was assessed by the Expanded Prostate Index Composite (EPIC) survey, and genitourinary (GU) and gastrointestinal (GI) toxicity were prospectively recorded (CTC v4.0). Global QOL scores were assessed for decline greater than the minimum clinically important difference (MCID). Univariate analysis (UVA) was performed, with 30-day post-implant dosimetry covariates stratified into quartiles. Median follow-up was 63 mo. Results: Men with NCCN low (*n* = 42) or favorable-intermediate risk (*n* = 37) disease were treated with brachytherapy alone, while most with high-risk disease had combined EBRT (*n* = 17 of 18). Men with UIR disease (*n* = 52) were selected for monotherapy (*n* = 42) based on clinical factors and MRI findings. Freedom from biochemical failure-7 yr was 98%. Of 37 men with MRI treated with monotherapy for UIR disease, all 36 men without extraprostatic extension were controlled. Late Grade 2+/3+ toxicity occurred in 55/3% for GU and 8/2% for GI, respectively. Fifty men were sexually active at baseline and had 2 yr sexual data; 37 (74%) remained active at 2 yr. Global scores for urinary incontinence (UC), urinary irritation/obstruction (UIO), bowel function, and sexual function (SF) showed decreases greater than the MCID (*p* < 0.05) in UC at 2 mo, UIO at 2 and 6 mo, and SF at 2–24 mo, and >5 yr. Analysis did not reveal any significant associations with any examined rectal or urethral dosimetry for late toxicity or QOL. Conclusion: Disease outcomes and patient-reported QOL support LDR brachytherapy, including monotherapy for UIR disease.

## 1. Introduction

Men with localized prostate cancer may be counseled to consider a variety of management options, including active surveillance, radical prostatectomy, or radiation therapy. The decision-making process ideally considers a patient’s disease risk, medical comorbidity, and preference. In many cases, especially in the setting of more favorable risk disease diagnosed in older men with competing risks of mortality, the benefits of therapy may not clearly outweigh the risks. Detailed knowledge regarding quality of life (QOL) outcomes can offer critical information to facilitate informed decisions [1] and minimize treatment related regret [2].

Brachytherapy, inclusive of both low-dose rate (LDR) and high-dose rate (HDR) brachytherapy, is a well-established treatment for select men with prostate cancer [3]. Prostate brachytherapy entails a procedure during which sealed radioactive sources are placed directly into the prostate, either via a permanent implant (LDR; often colloquially referred to as radioactive seeds) or temporarily via an interstitial catheter (HDR). The placement of the radioactive source directly into the prostate confers the ability to deliver a high radiation dose to the target and limit the exposure of adjacent normal tissue with a relatively steep dose gradient in comparison to external beam radiation therapy (EBRT). Some men with prostate cancer can be treated with brachytherapy as monotherapy, while others are treated with a combination of EBRT followed by an implant boost, given the possibility for subclinical involvement of the seminal vesicles or lymph nodes. In men with intermediate risk disease, this decision is typically made based on clinical factors, such as the widely accepted risk classification of favorable intermediate risk (FIR) versus unfavorable intermediate risk (UIR) disease supported by the NCCN [4]. While the efficacy of monotherapy for FIR disease is generally accepted [5], the management of men with UIR disease is less certain. Offering men supplemental EBRT to treat the seminal vesicles or lymph nodes can potentially improve outcomes [6,7] but also carries the risk of increased toxicity. Guidelines currently recommend monotherapy for only low- or favorable intermediate-risk (FIR) [4,8].

In this study, we analyzed our 15-year institutional experience of low dose rate (LDR) brachytherapy for prostate cancer to evaluate two specific topics. First, we sought to contribute to the knowledge gap regarding the efficacy of brachytherapy monotherapy in unfavorable intermediate risk (UIR) disease, reporting on the value of pre-treatment MRI that was routinely incorporated into decision making. Secondly, we wished to report detailed toxicity and QOL outcomes and explore whether post-implant dosimetry were correlated, hoping that a more contemporary analysis may offer new insights to implant guidelines that have changed little since 1999 [8,9,10,11]. Although many comparative QOL outcomes with LDR brachytherapy as monotherapy have been reported [1,12,13,14,15], these studies may be limited by a lack of detailed patient and treatment factors, or by a relatively shorter follow-up.

## 2. Materials and Methods

Men diagnosed with non-metastatic prostate cancer who were treated with brachytherapy between 2006–2021 at one institution by a single radiation oncologist were enrolled in a prospective, longitudinal study to analyze disease outcomes, toxicity, and post-treatment quality of life (IRB #14934A). All men underwent permanent implant Iodine-125 LDR brachytherapy, prescribed to 144–145 Gy as monotherapy or 110 Gy as a boost following external beam radiation therapy (EBRT, median 45 Gy) to the pelvic lymph nodes [16]. Brachytherapy was offered as a treatment choice for eligible candidates following a consistent treatment algorithm over the period of study (Supplemental Figure S1). Candidacy for brachytherapy included the lack of severe urinary dysfunction (International Prostate Symptom Score < 15) or larger prostate gland size (<60 cc), and no prior history of transurethral resection of the prostate. The majority of men (*n* = 114) underwent endorectal MRI prior to brachytherapy; this imaging test was more commonly ordered in men with intermediate risk disease (80/89, 90%) than in men with low risk (23/42, 55%) or high risk (11/18, 61%) disease. Androgen deprivation therapy (ADT) was prescribed for two indications: men treated with monotherapy with an enlarged prostate size (e.g., size > 60 cc, or largest transverse dimension of >5 cm) received oral only therapy consisting of a 5-alpha reductase inhibitor with bicalutamide for gland downsizing; men treated with brachytherapy boost after EBRT were prescribed a luteinizing hormone-releasing hormone to augment treatment efficacy [17]. A combined EBRT and brachytherapy boost was reserved for men with higher risk features that increased the risk of pelvic nodal involvement at the clinician’s discretion. Generally, this applied to nearly all men with NCCN high-risk prostate cancer and select men with NCCN UIR disease (multiple cores of primary Gleason pattern 4, PSA > 15, percent positive biopsy cores >67%, or MRI with high-risk findings on staging endorectal MRI [17,18] such as gross extraprostatic extension (EPE), seminal vesicle invasion, or lymph node involvement). For the purposes of this study, EPE was recorded on a 4-point scale of definitely negative (grade 0), probably negative (grade 1), indeterminate (grade 2), probably positive (grade 3), and definitely positive (grade 4) based on interpretation by a genitourinary radiologist, as described previously [18].

Brachytherapy was delivered using a pre-plan approach, with 18 Gauge needles preloaded with stranded I-125 seeds (IsoAid LLC, Port Richey, FL, USA). Two to three weeks prior to the seed implant, all patients underwent a transrectal ultrasound volume study. After a bowel prep including oral simethicone and an enema, the patient was positioned supine in the dorsal lithotomy position with both legs raised with knees flexed at 90 degrees. A urinary catheter was used to drain and fill the bladder with 120 cc saline before attaching a syringe with aerated KY jelly to highlight the urethra. Axial ultrasound images were acquired every 5 mm from the base to the apex of the gland. Images were then transferred to a dedicated prostate brachytherapy software (Variseed v7.6–9.0, Varian Medical System, Palo Alto, CA, USA) for treatment planning. Once the prostate volume was delineated, a planning target expansion of 0–5 mm was applied to the prostate, including 0 mm posteriorly at the mid-gland, 2 mm anteriorly and laterally, and up to 5 mm circumferentially at the base and apex. Pre-implant planning goals included PTV V100 > 98%, V150 < 40%, V200 < 20%, and mean urethral dose <120% of the prescription dose. The implantation of radioactive seeds was performed jointly with a urologist in an operating suite. All patients were under general anesthesia with paralysis, unless contraindicated in favor of spinal anesthesia. A transrectal ultrasound probe (from 2006–2020 Hitachi 5500; from 2021 onwards, BK3000) was affixed to a stepper and stabilizer system (CIVCO). The seed placement was performed using a grid template registered to the probe, guided by the real time ultrasound images and fluoroscopy as needed for accurate placement. Cystoscopy was not routinely performed at the conclusion of the procedure. All men were prescribed an alpha-blocker prophylactically prior to implant to aid with urination (e.g., terazosin 5 mg or tamsulosin 0.4 mg). CT-based post implant dosimetry was performed at day 30, as recommended by the AAPM Task group 137 [19]. Axial CT images in 2 mm slice thickness were acquired on a Bigbore scanner (Philips, The Netherland). All images were transferred to the same software (Variseed) for prostate, urethra and rectum delineation. Seed localization and dose volume calculations were subsequently performed by qualified medical physicists. The CT prostate volumes were determined with a visual correlation to pre-implant ultrasound volumes to ensure similar total volume. The urethra was contoured based on the visual correlation of the pre-implant position, without placement of a urinary catheter or contrast dye. The rectum was contoured at the level of the prostate. The dosimetry quality of the implant was assessed using the ABS guidelines for a permanent prostate seed implant, i.e., prostate V100 > 85%, D90 > 90%, and rectal V100 < 1 cc [8].

Follow-up consisted of a clinical exam, the evaluation of PSA, and patient-reported QOL every 6–12 months up to 10 years after the implant. Freedom from biochemical failure (FFBF) was defined as a PSA that was no more than 2 ng/mL above the nadir, excluding any transient rises in PSA that subsequently decreased without therapy. QOL was evaluated by the Expanded Prostate Index Composite (EPIC) and International Prostate Symptom Score (IPSS), and physician-assessed genitourinary (GU) and gastrointestinal (GI) toxicity scores (CTC v4.0) were prospectively assigned at each visit. For urinary frequency, the definition of late grade 2+ toxicity included any use of new or increased dose of urinary medication (e.g., tamsulosin) to aid with frequency beyond 3 months. The study was pragmatic in design in order to allow patients to alternate visits between specialists and grant flexibility in the timing of QOL surveys, which were recorded for radiation oncology visits.

Global QOL scores were assessed for decline greater than the minimum clinically important difference (MCID) [20]. QOL endpoints were assessed with a multivariate repeated measures ANOVA using PROC MIXED in SAS (version 9.4, SAS Institute, Cary, NC, USA) to avoid the listwise deletion of missing data. QOL at “5+ yr” included the most recent data beyond 5 yr. Overall survival and freedom from toxicity were assessed with the Kaplan-Meier method in JMP (version 14, SAS Institute, Cary, NC, USA).; the log-rank test statistic assessed differences in levels of selected prognostic factors including dosimetric parameters. Univariate analysis (UVA) for toxicity and QOL was performed, with post-implant dosimetry covariates stratified into quartiles. QOL endpoints were assessed with a multivariate repeated measures ANOVA.

## 3. Results

A total of 149 men with prostate cancer received LDR brachytherapy, including 122 (82%) as monotherapy and 27 (18%) in combination with EBRT. Patient and treatment characteristics are shown in Table 1. The median pre-treatment PSA was 6.2, and age was 64 yr. The Gleason score was 6 (32%), 7 (57%), or 8–9 (10%). Men with NCCN low (*n* = 42, 28%) or FIR (*n* = 37, 25%) disease were treated with brachytherapy alone. Most men with UIR disease (*n* = 52, 35%) had monotherapy (*n* = 42; combined EBRT *n* = 10), while most with high-risk disease (*n* = 18, 12%) had combined EBRT (*n* = 17). Forty-seven men had androgen deprivation therapy (median 6 mo). Downsizing for a large gland was typically achieved using oral only agents (*n* = 18; e.g., finasteride and bicalutamide for 3 months), whereas combined hormonal therapy to improve disease outcomes otherwise included an LHRH agonist (*n* = 29).

In 114 men with staging MRI, probable or definite EPE was observed in 0 of 23 (0%) men with low-risk, 3 of 34 (9%) men with FIR, 5 of 46 (11%) men with UIR, and 6 of 11 (55%) men with high-risk disease. Seminal vesicle invasion (*n* = 1) or lymph node involvement (*n* = 0) were rarely observed (<1%). The largest dominant nodule seen on MRI measured a median 10 mm (IQR 7–14 mm).

Post-implant dosimetry was performed at a median of 30 days. The median D90 prostate, V100 prostate, rectal V100%, and mean urethral dose were 101%, 91%, 0.12 cc, and 165 Gy, respectively. The majority of cases met the ABS post-implant goals for prostate V100 ≥ 85% (*n* = 126, 85%), prostate D90 ≥ 90% (*n* = 143, 96%), and rectal V100 < 1 cc (*n* = 140, 94%).

With a median follow-up of 63 mo, FFBF at 5 years was 100%, and at 7 years it was 98%. Overall, 3 men experienced biochemical failure, including one man with low-risk disease (at 176 mo, with biopsy proven distant metastasis to para-aortic nodes) and 2 with UIR disease (at 62 and 115 mo, biochemical failure without clear local recurrence or distant metastasis, treated with salvage hormonal therapy). Four men experienced a bounce in PSA >2 ng/mL that resolved without therapy and were not included as biochemical failures; overall, 38 men had a transient rise in PSA after brachytherapy that was not related to testosterone recovery after ADT (median 0.28, IQR 0.14–1.29, range 0.02–4.4 ng/mL; median time to PSA bounce 21 months). In 37 men with pre-treatment MRI available who were treated for UIR disease with monotherapy, all 36 men without EPE were biochemically controlled at a median follow-up of 49 months, while the only man with probable EPE experienced biochemical failure at 62 months (*p* = 0.0002). Patient characteristics, MRI findings, and disease outcomes for men with UIR disease are shown in Table 2. The 7-year FFBF for all 42 men with UIR disease treated with monotherapy was 95%; the 7-year FFBF for all men otherwise treated with monotherapy for low-risk (*n* = 42), FIR (*n* = 37), and high-risk disease (*n* = 1) was 100%, and was likewise 100% for men with UIR (*n* = 10) or high-risk (*n* = 17) disease treated with brachytherapy boost. Median PSA at last follow-up was 0.17. In men with >4 years of PSA follow-up, the median PSA at last follow-up was undetectable (<0.05 ng/mL) in 31 of 58 (53%) men. At the time of last follow-up, 14 men were deceased, with none of three biochemical recurrences with castrate sensitive disease at the time of death attributed to prostate cancer. Secondary cancers were observed in 10 men; the only cancer in proximity to the prostate was a T1N0 bladder cancer with a history of tobacco and asbestos exposure diagnosed 2 years after implant, successfully treated with a radical cystectomy.

Acute urinary obstruction within 6 months of the implant occurred in 10 (7%) men, and was managed with a temporary Foley catheter (*n* = 8, median 6 days) or suprapubic tube (*n* = 2, median 3 mo). Late Grade 2+ and 3+ GU toxicity was observed in 55% and 3% (in five men including urinary obstruction requiring a temporary Foley catheter (*n* = 1 at 20 mo) or TURP (*n* = 2, at 12 and 19 mo), and hematuria requiring a temporary Foley catheter (*n* = 1, at 63 mo) or cystectomy related to fistula repair in the setting of nephrolithiasis and urologic instrumentation (*n* = 1, at 9 yr). Late grade 2+ and 3+ GI toxicity in 8% and 2% (*n* = 3, including two with endoscopic coagulation of proctitis and one with intermittent fecal urge incontinence requiring pad use), respectively. The high incidence of cumulative grade 2+ GU toxicity was primarily attributable to the frequent use of urinary medicines within six months of brachytherapy; urinary medicines were documented at last follow-up in 37%. There was an increase in IPSS over baseline of a median 9, 3, and 2 points at 2 mo, 1 yr, and 5+ yr, respectively (all *p* < 0.01). Among 77 men with baseline and 2-year data on sexual activity, 50 (65%) were active at baseline, and 37 of those (74%) remained active at 2-years.

Global scores for urinary incontinence (UC), urinary irritation/obstruction (UIO), bowel function, and sexual function (SF) over time are shown in Supplemental Table S1. Decreases greater than the MCID (*p* < 0.05) were observed in UC at 2 mo (−19, *p* = 0.02), UIO at 2 mo (−25, *p* < 0.01) and 6 mo (−12, *p* = 0.02), and SF at 2–24 mo (−30 at 2 mo, *p* < 0.01; −17 at 24 mo, *p* = 0.04). Analysis did not reveal any significant associations with any examined patient or treatment factors or post-implant dosimetry with late grade 2–3+ toxicity (Table 3) or QOL.

QOL after brachytherapy over time by symptom is shown in Table 4. Outcomes for monotherapy versus brachytherapy boost were consolidated into one table because univariate and multivariate analysis suggested no difference in QOL between the two groups. At 5+ yr, the percentage of men reporting moderate/severe distress for overall urinary, bowel, and sexual function (8%, 4%, and 22%) was numerically similar to baseline (10%, 3%, 23%), respectively.

## 4. Discussion

This consecutive cohort of 149 men treated with LDR brachytherapy for prostate cancer revealed excellent biochemical outcomes, including those with UIR disease treated with monotherapy. Brachytherapy patients had reasonably well preserved QOL, with transient changes in acute urinary QOL, and longer-term changes in sexual QOL greater than the MCID. Post-implant dosimetry did not correlate with toxicity or QOL. The patient-reported symptom distress table may assist men who are evaluating treatment options to guide expectations and make an informed, personal choice.

Overall, our results are generally consistent with published literature on prostate brachytherapy, including treatment efficacy [21,22], impact on quality of life [1,23], and a lack of correlation with dosimetry on toxicity or quality of life beyond the few ABS endorsed of D90 to the prostate and rectal V100 [7]. This consistency in outcome should perhaps come as no surprise since the pre-plan approach to radioactive seed implant has been widely adopted and we describe an experience in the context of the well-controlled environment of a single institution and provider. The inability to uncover new correlations with post-implant dosimetry and outcome might also be expected, especially considering our sample size, low number of events, and potential inability for 30-day assessment using the relatively crude tools of urethral and rectal dose to reflect pathology over the life of the implant. In this context, ABS endorsed post-implant goals are important parameters for programs to strive to achieve, but at the patient level may not offer a numerically absolute form of assessing implant adequacy in which any slight deviation could risk a compromise in outcome.

There are two potential areas where our work contributes new information on LDR prostate brachytherapy. First, we demonstrated a very high rate of disease control (5-yr FFBF 100%; 7-yr FFBF 98%) with brachytherapy within the construct of a treatment algorithm that routinely incorporated staging endorectal MRI for intermediate risk disease. Over the duration of the study, this imaging test was ordered for risk stratification–men without gross extraprostatic extension, seminal vesicle invasion, or lymph node involvement [18] were commonly offered monotherapy, even if they presented with UIR disease. The excellent disease control of the UIR cohort (7-yr FFBF 94%) supports the rationale to offer brachytherapy monotherapy for men without high-risk MRI findings. The RTOG 0232 randomized trial of brachytherapy +/− partial pelvic EBRT for intermediate risk prostate cancer showed no difference in 5-y progression free survival (85–86% in both arms) [5], but men with UIR disease were under-represented, and a subset analysis on this group has not yet been reported. Given the lack of evidence in this area, recently published guidelines by the NCCN [4] and the ABS [8] favor the use of EBRT + brachytherapy rather than brachytherapy alone for these men. These guidelines are consistent with data from an NCDB analysis in which men with UIR disease treated with supplemental EBRT had improved survival compared to those treated with brachytherapy alone6, although other institutional studies suggest no impact of additional EBRT on survival [24,25,26]. Extending brachytherapy monotherapy as an effective option for UIR disease, which has a disease biology similar to high-risk disease [27], would potentially reduce late grade 2–3 toxicity and improve QOL [28] by eliminating the overtreatment of the pelvis with EBRT.

Second, we provide patient-reported quality of life in the more user-friendly format of a symptom distress table, in order to complement EPIC global domain scores. Table 4 is intended to be used as a tool among clinicians and patients to frame expectations of specific symptoms over time, which can be discussed in the context of other treatment modalities, similar to the 2-year outcomes from the PROST-QA consortium1. We did not find further deterioration in patient reported urinary, bowel, or sexual quality of life beyond 2 years, but it is notable that other long-term studies have reported this possibility [29,30]. A few other trials and multi-institutional collaborations have published high quality reports regarding quality of life after prostate cancer therapy. One of the most detailed is a 5-year report from a prospective, population-based cohort study [12] showing unique quality of life changes over time for men with favorable intermediate risk prostate cancer treated with various approaches. In 86 men treated with LDR brachytherapy, clinically meaningful decreases in quality of life were observed with urinary incontinence, urinary irritative, bowel, and sexual function when compared to men on active surveillance, although none of these differences persisted beyond 1 year. In contrast, men undergoing radical prostatectomy reported longer term changes including inferior sexual function (3 yr) and urinary incontinence (5 yr), whereas men treated with EBRT had no clinically meaningful decrease in any measured quality of life domain at any time point when compared to surveillance. In this context, brachytherapy carries more risk to near-term quality of life than EBRT, which could be justified by its unique benefits, including the ability to escalate radiation doses and limit low doses of scatter radiation when compared to EBRT.

The utilization of brachytherapy for prostate cancer has decreased in the United States from a peak 20 years ago [31]; this is attributable to several factors, including the increasing acceptance of active surveillance, a more extensive menu of competing therapeutic options (including robotic prostatectomy, intensity modulated radiation therapy, and proton therapy), and, more recently, the proven efficacy and safety of moderately hypofractioned radiation therapy. Stereotactic body radiation therapy [32], as well as simultaneous integrated nodule boosts [33], may further challenge the use of brachytherapy, as these “nearly-ablative” dose escalation options could offer a better benefit/risk ratio for some patients compared to the higher doses prescribed in brachytherapy. In our clinical practice (Supplemental Figure S1), prostate brachytherapy may add value in select areas to complement the wide range of EBRT indications: (1) it is an excellent alternative to EBRT or surgery that some men may prefer, considering the relative QOL outcomes [1,12,14]; (2) men with longer life expectancy (e.g., >15 years) may benefit from the improvement in biochemical control over EBRT achieved with the ablative doses of brachytherapy [34,35,36,37] to reduce the need for salvage therapy after progression; (3) some men at substantial risk for treatment failure, especially if it is more likely to be local than distant (e.g., moderate to high volume, Gleason score ≤8 disease), may be better served to accept the QOL risks of brachytherapy boost rather than those of long term hormonal therapy with abiraterone [38] when choosing a form of treatment intensification. Ideally, the best choice for any patient will involve shared decision making, and acknowledge the unique logistics and risks of brachytherapy, including an increased risk of late irritative /obstructive urinary side effects.

Although this report is limited to LDR brachytherapy, HDR brachytherapy is another effective option available for the treatment of prostate cancer. Notable differences exist with regard to the logistical planning and near-term side effect profile between these approaches, but outcomes reveal similar long-term efficacy, toxicity, and quality of life3. Our institution has not chosen to convert from LDR to HDR brachytherapy for prostate cancer, as the benefit/risk ratio has been perceived to be favorable, and the HDR approach may place more demands on patient and provider time including anesthesia, and requires more advanced care coordination (e.g., radiation planning, hospital admission, and multiple implants required for patients treated with monotherapy). However, there are likely to be advantages with regard to radiation dosimetry, time to resolution of acute side effects, and decreased clinician/staff exposure to ionizing radiation; moreover, HDR brachytherapy obviates the need for radiation precautions to be observed at the time of discharge. Practices can make informed decisions on which form of brachytherapy is more suitable when considering the needs of a particular patient population and the staffing and capabilities of an individual program.

The limitations of this study include the single-institution, non-randomized design, with patients following a median of 63 months. In contrast to the multi-institutional, randomized trial RTOG 0232, our series demonstrated a higher rate of 5-year PSA control, and no detriment of combination EBRT on toxicity or QOL, which could potentially be due to differences in follow-up length and completeness. As an institutional study in which all men were treated by a single physician, our results are less certain to translate to all practices. However, we used a defined treatment algorithm routinely incorporating endorectal MRI and followed the standard technical approach of LDR brachytherapy that became widely practiced more than 20 years ago [21]. Our results may also be limited by the sporadic nature of QOL survey completion follow-up due to the pragmatic approach in obtaining the QOL data, as many patients completed surveys only once yearly. The attrition in follow-up may also have limited the chance to reveal associations in post-implant dosimetry with toxicity or QOL. Finally, pre-treatment MRI was interpreted by specialized genitourinary radiologists for the presence of EPE, which may be subject to the experience of the reader [39,40], although with practice, discussion with colleagues in radiology, and the use of illustrative tools available [41], it is reasonable to expect a radiation oncologist to improve their fluency in image interpretation.

In summary, the disease and quality of life outcomes shown in this study support the utilization of LDR brachytherapy for prostate cancer as described in our institutional treatment paradigm. Pre-treatment MRI, by identification of extraprostatic extension, is capable of refining selection for men with UIR disease who may be candidates for monotherapy. Further study is necessary to discover patient or treatment factors which correlate with increased toxicity or inferior QOL in an effort to further tailor the wide range of radiotherapeutic treatments available according to the needs of the patient.

## Figures and Tables

**Table 1:** Patient and treatment characteristics.

	Median (IQR) or Number (%)
Age (years)	Median 64 (59–69)
Race (*n* = 148)	
Caucasian	61 (41%)
African American	77 (52%)
Hispanic	6 (4%)
Asian	3 (2%)
Other	1 (1%)
PSA at Diagnosis (ng/mL)	6.2 (4.8–8.9)
Gleason Score	
Gleason 3 + 3 (grade group 1)	49 (33%)
Gleason 3 + 4 (grade group 2)	70 (47%)
Gleason 4 + 3 (grade group 3)	15 (10%)
Gleason 4 + 4 (grade group 4)	7 (5%)
Gleason 9–10 (grade group 5)	8 (5%)
Clinical stage	
T1c	117 (78%)
T2a	18 (12%)
T2b	7 (5%)
T2c	2 (1%)
T3a	5 (3%)
Percent biopsy cores positive	33 (21–50)
NCCN Risk grouping	
Low	42 (28%)
Favorable-intermediate risk	37 (25%)
Unfavorable-intermediate risk	52 (35%)
High	18 (12%)
Brachytherapy dose	
Monotherapy, 144 Gy	15 (10%)
Monotherapy, 145 Gy	107 (72%)
Combined with external beam, 110 Gy	27 (18%)
Hormonal therapy	
Oral agents only	18 (13%)
LHRH agonist	29 (19%)
Duration (mo)	6 (3–24)
Post Implant Dosimetry	
V100 prostate (%)	91 (87–94)
V150 prostate (%)	37 (32–43)
V200 prostate (%)	13 (11–16)
V100 Rectum (cc)	0.12 (0.02–0.39)
D1cc Rectum (Gy)	94.8 (69.1–110.3)
Mean urethra dose (Gy)	165 (148–183)
Median Follow-Up (months)	63 (28–110)

**Table 2:** Patient characteristics, MRI findings, and outcomes for men with unfavorable intermediate risk disease (*n* = 52).

	Monotherapy Median (Range) or Number (%)	Brachytherapy Boost Median (Range) or Number (%)	*p* Value
Age (years)	65.5 (42–77)	63 (53–71)	0.66
PSA at Diagnosis (ng/mL)	6.15 (1.9–14.5)	8.35 (4.5–19.0)	0.0578
Gleason Score			0.40
Gleason 3 + 4 (grade group 2)	31 (74%)	6 (60%)	
Gleason 4 + 3 (grade group 3)	11 (26%)	4 (40%)	
Clinical stage			0.36
T1c	38 (83%)	8 (80%)	
T2a	2 (5%)	0 (0%)	
T2b	2 (5%)	2 (20%)	
T2c	2 (1%)	0 (0%)	
Percent biopsy cores positive	50 (8–100)	64 (36–94)	0.0719
MRI findings (*n* = 46)			
EPE grade (0/1/2/3/4)	25/1/10/1/0	3/1/1/1/3	0.0068
EPE probable or definite	1 (3%)	4 (44%)	0.86
Largest nodule axial dimension, mm	11 (4–20)	11 (6–27)	0.0098
Hormonal therapy			<0.0001
Oral agents only	3 (7%)	1 (10%)	
LHRH agonist	1 (2%)	9 (90%)	
Duration (mo)	3 (3–4)	6 (2–28)	
7-year FFBF			
Overall group	94%	100%	0.74
Men without EPE on MRI	100%	100%	NA
Median PSA Follow-Up (months)	49 (4–136)	34 (11–90)	0.20

EPE = Extraprostatic extension; FFBF = freedom from biochemical failure.

**Table 3:** Univariate analysis of various covariates against late toxicity.

Variable	Freedom from GI 2+ Toxicity	Freedom from GI 3+ Toxicity	Freedom from GU 2+ Toxicity	Freedom from GU 3+ Toxicity
Age (< vs. >= 67)	0.6174	0.6162	0.4159	0.3145
Combined EBRT	0.0829	0.577	0.1973	0.4339
Anticoagulation	0.8363	0.9948	0.6571	0.1421
Diabetes	0.5810	0.6079	0.6350	0.5749
Rectal D1cc (Gy)	0.5125	0.8596		
Rectal V100 (cc)	0.5625	0.6180		
Mean urethra dose (Gy)			0.1453	0.6236
Urethra D.01cc (Gy)			0.4604	0.6484
Urethra D1 (%)			0.1881	0.7652
Urethra V125 (cc)			0.5886	0.7324
Urethra V125 (%)			0.4667	0.1330
Urethra V150 (cc)			0.8302	0.6325
Urethra V150 (%)			0.6793	0.4552
Urethra V200 (Gy)			0.4618	0.6159

*p* values shown. EBRT = external beam RT.

**Table 4:** Symptom distress over time.

		Baseline *n* = 123	3 mo *n* = 26	6 mo *n* = 64	1 yr *n* = 87	2 yr *n* = 93	3 yr *n* = 64	4 yr *n* = 51	5+ yr *n* = 51
**Urinary function**									
	Dysuria	1%	14%	5%	7%	4%	0%	0%	0%
	Hematuria	1%	0%	2%	0%	0%	0%	0%	2%
	Weak stream	2%	33%	22%	11%	9%	7%	0%	4%
	Frequency	12%	26%	28%	19%	19%	14%	10%	8%
**Urinary incontinence**									
	Leaking >1 time per day	3%	13%	9%	4%	6%	5%	2%	8%
	Frequent dribbling	4%	29%	14%	1%	6%	6%	0%	4%
	Any pad use	2%	17%	6%	5%	4%	5%	4%	8%
	Leaking problem	2%	13%	6%	1%	2%	3%	0%	4%
**Overall urinary problem**		10%	46%	16%	13%	15%	6%	4%	8%
**Bowel function**									
	Urgency	8%	20%	16%	6%	9%	9%	8%	0%
	Frequency	2%	13%	9%	5%	3%	2%	2%	0%
	Fecal incontinence	0%	0%	8%	1%	1%	2%	2%	2%
	Bloody stools	1%	4%	3%	2%	2%	6%	2%	2%
	Rectal pain	2%	9%	3%	2%	0%	2%	2%	2%
	Diarrhea	7%	12%	19%	12%	11%	8%	6%	10%
**Overall bowel problem**		3%	8%	11%	0%	3%	3%	4%	4%
**Sexual function**									
	Poor erections	23%	63%	44%	47%	46%	42%	30%	41%
	Difficulty with orgasm	17%	58%	43%	38%	31%	33%	18%	30%
	Erections not firm	18%	56%	40%	30%	33%	33%	22%	33%
	Erections not reliable	30%	74%	55%	48%	42%	39%	37%	34%
	Poor sexual function	27%	73%	52%	47%	46%	44%	33%	40%
	Sexually active	59%	12%	38%	55%	54%	58%	59%	49%
**Overall sexual problem**		23%	46%	43%	39%	31%	32%	27%	22%

## Data Availability

To ensure compliance with the existing IRB approval letter and HIPAA compliance for patients, the full de-identified dataset cannot be made available without prior written approval from the University of Chicago Hospital IRB. Requests for de-identified data can be made to the corresponding author.

## Supplementary Materials

The following supporting information can be downloaded at: https://www.mdpi.com/article/10.3390/cancers15041336/s1, Table S1: Global quality of life over time after LDR brachytherapy. Figure S1: Institutional treatment paradigm at the University of Chicago for prostate cancer management.
