# Supplementary material for: A Contemporary Report of Low-Dose-Rate Brachytherapy for Prostate Cancer Using MRI for Risk Stratification: Disease Outcomes and Patient-Reported Quality of Life
Source: Cancers (Basel). 2023 Feb 20;15(4):1336. doi: 10.3390/cancers15041336 (PMC9953871; doi:10.3390/cancers15041336)
Supplement: Supplementary file 1 [file cancers-15-01336-s001.zip › cancers-2186075-supplementary.pdf]

Supplemental Table 1. Global quality of life over time after LDR brachytherapy.

	Urinary Incontinence				Urinary Irritation				Bowel				Sexual			
	N	Med	Min	Max	N	Med	Min	Max	N	Med	Min	Max	N	Med	Min	Max
Baseline	121	100	42	100	114	94	50	100	122	100	46	100	116	74.5	0	100
3 months	22	77	25	100	20	66	38	100	24	93.5	46	100	24	23	0	88
6 months	62	86	31	100	61	81	31	100	64	96	21	100	61	49	0	100
12 months	83	100	50	100	82	88	31	100	85	96	58	100	84	51	0	100
24 months	93	100	40	100	93	88	38	100	93	100	46	100	87	57	0	100
36 months	62	100	17	100	58	94	50	100	63	100	58	100	62	60.5	0	100
48 months	48	100	61	100	47	94	63	100	51	100	25	100	49	63	0	92
60+ months	50	100	40	100	51	94	63	100	49	100	42	100	50	54	0	100

Supplemental Figure S1. Institutional treatment paradigm at the University of Chicago for prostate cancer management. Brachytherapy was prioritized over external beam radiation for eligible candidates with life expectancy >15 years. Clinical factors such as MRI were used to stratify patients with favorable or unfavorable intermediate risk disease, informing the decision regarding brachytherapy alone or as a supplement to initial external beam. This treatment paradigm represented general institutional practice over a significant portion of the study period, but should not be used in favor of the most current, evidence-based guidelines, such as endorsed by the NCCN.

Institutional Treatment Paradigm for Intact Prostate Cancer

Assess candidacy for radiation therapy, goals of therapy, and patient comorbidity

Discuss: Treatment vs. surveillance, surgery vs. RT

NCCN risk category:	Low-risk	Intermediate risk		High risk	
		Favorable (e.g. no higher risk MRI findings)	Unfavorable (e.g. higher risk MRI findings)	T2; ≤GS8; lower volume	T3; GS9-10; N1; higher volume
External Beam RT:	Prostate only	Prostate/SV	Prostate/SV	Prostate/SV	Pelvic LN, Prostate/SV
Hormonal therapy:	No	No	6 mo	6 vs. 18+ mo	18+ mo; +/- abiraterone
Brachytherapy*:	Monotherapy	Monotherapy	Boost after EBRT	Boost after EBRT	Boost after EBRT

**Priority over EBRT if life expectancy >15 yr*

- Medical comorbidity can downstage treatment intensity
- Consider protocol enrollment a priority when eligible
